# Postponed childbearing: a cross-sectional study of differences between subjective and objective factors

**DOI:** 10.1080/07853890.2025.2546674

**Published:** 2025-08-27

**Authors:** Xin Li, Siyuan Zeng, Yalan Li, Liling Xiong, Yaoyao Zhang, Juan Zou, Changsheng Lin, Lei Yu, Peina Yang, Ting Hu, Xue Xiao, Tianjiao Liu

**Affiliations:** ^a^ West China Second University Hospital, Sichuan University, Chengdu, China; ^b^ Key Laboratory of Birth Defects and Related Diseases of Women and Children (Sichuan University), Ministry of Education, West China Second Hospital, Sichuan University, Chengdu, China; ^c^ Medical Genetics Department, West China Second University Hospital Sichuan University China, Chengdu, China; ^d^ The Fourth People’s Hospital of Chengdu, School of Medicine, University of Electronic Science and Technology of China, Chengdu, China; ^e^ Medical Genetics Department, Sichuan University West China Second University Hospital Department of Medical Genetics, Chengdu, China; ^f^ Department of Gynaecology and Obstetrics, Chengdu Women’s and Children’s Central Hospital, Chengdu, China

**Keywords:** Career prioritization, objective factors, postponed childbearing, subjective factors

## Abstract

**Background:**

To investigate the factors influencing postponed childbearing in women, focusing on the differences between subjective and objective factors.

**Methods:**

This study included 1,128 women who were recruited from three hospitals between January and December 2023. Participants with a prior history of mental health disorders or those who chose not to participate were excluded from the study. Data were collected through early pregnancy prenatal checkups and questionnaires. Binary logistic regression was used to analyze the impact of both subjective factors and objective factors on postponed childbearing.

**Results:**

Of the 1128 participants, 507 were in the normal childbearing group and 621 were in the postponed childbearing group. The study found that higher pre-pregnancy body mass index, higher educational level, full-time employment, and reproductive health issues were independently associated with an increased likelihood of postponed childbearing. From a subjective perspective, the primary factors influencing the decision to delay childbirth were career prioritization and financial stability. Women who placed greater importance on their professional aspirations were 72% more likely to postpone having children, while those who expressed concerns regarding financial security had a 128% increased probability of doing so.

**Conclusion:**

Postponed childbearing is influenced by a combination of subjective perceptions and objective factors. Socio-economic status, career goals, and psychological influences, all contribute significantly to the choice of postponing childbearing. These findings highlight the importance of addressing both the health and socio-economic challenges faced by women, emphasizing the need for policies that support women in balancing career, family, and reproductive health.

## Introduction

In recent decades, the trend of postponed childbearing has become a prominent phenomenon in many parts of the world, particularly in developed countries [1,2]. Women are increasingly choosing to postpone childbirth for a variety of reasons, including educational and career aspirations, economic pressures, and shifting social norms regarding marriage and family life [3]. As a result, the average age of women at the time of their first pregnancy has gradually increased. This delay in reproductive age is not only a significant sociological and cultural shift but also a public health concern due to its potential implications for maternal and child health [4,5]. Given the increasing prevalence of postponed childbearing and its multifaceted consequences, understanding the underlying factors is crucial for informing public health strategies and supporting women’s reproductive choices.

Several factors contribute to the decision to delay childbearing. Educational attainment has been shown to be a key determinant, with women pursuing higher levels of education often delaying marriage and childbearing to focus on career development [6,7]. Economic factors, such as job instability, the high cost of living, and the desire for financial security, also play a crucial role in influencing women’s decisions regarding when to have children [8,9]. Additionally, the evolving societal expectations surrounding marriage and family dynamics—where women now have greater autonomy in reproductive decisions—has further contributed to the trend of postponed childbirth [10]. Emerging research also highlights the importance of psychological influences, such as perceived readiness, anxiety over reproductive aging, and social expectations, which can shape women’s attitudes toward the timing of childbirth.

While the delay in childbearing may offer women more control over their life trajectories, it is not without its risks. Biologically, fertility declines with age, particularly after the age of 35 [11,12]. Older maternal age has been associated with increased risks of pregnancy-related complications, such as gestational diabetes, preeclampsia, and chromosomal abnormalities, including Down syndrome [13,14]. Furthermore, women who delay childbirth often experience a reduced ovarian reserve, leading to challenges in conception even with assisted reproductive technologies [15]. Consequently, the shift towards later childbearing has raised concerns about the long-term health implications for both mothers and their children.

In addition to the health risks, the postponed age of motherhood has broader societal and economic consequences. Older maternal age can affect population demographics, with fewer women having children at younger ages, potentially contributing to an aging population [16]. This demographic shift poses challenges for public health systems, as healthcare services must adapt to meet the needs of an aging population while still addressing the complexities of maternal and neonatal care. Furthermore, postponed childbearing can influence the development of future generations, as research suggests that the age of the mother at the time of birth may impact the child’s cognitive development, health outcomes, and long-term well-being [17,18].

Given the importance of understanding the socio-demographic and psychological factors that contribute to postponed childbearing, this study seeks to analyze the patterns and determinants of postponed reproductive age among women seeking prenatal care at hospital. This research seeks to offer an in-depth understanding of the factors driving postponed childbearing and its impact on maternal and child health by analyzing socio-economic elements such as education, employment, marital status, and lifestyle habits, alongside subjective influences like career ambitions, financial stability, and psychological concerns.

## Materials and methods

### Study design and participants

This investigation utilized a cross-sectional methodology based on a hospital cohort approach. All eligible pregnant women in their early gestational stages who visited the hospital for their first prenatal appointment during the study period were consecutively recruited and administered structured questionnaires. The goal was to explore the socio-demographic variables linked to postponed childbearing and to examine the impact of socio-economic, cultural, and medical factors on women’s reproductive decisions. The study sample consisted of women attending the obstetrics and gynecology department of the hospital during their first trimester (up to 12 weeks of pregnancy) from January to December 2023. The study included women aged 18–45 years who were attending their initial prenatal consultation at the hospital. Exclusion criteria encompassed individuals with a prior history of mental health conditions and those who chose not to participate in the study. Within the field of reproductive medicine, women aged 30 years and older are often considered to have postponed childbearing, as such delays are associated with an elevated risk of adverse pregnancy outcomes [19,20]. Consequently, participants were categorized into two groups for this study: the ‘Normal childbearing group’ for those under 30 years and the ‘Postponed childbearing group’ for those over 30 years.

This study was approved by the Institutional Review Board (IRB) of Chengdu Women’s and Children’s Central Hospital (2023097). Informed consent was obtained from all participants before data collection. Participants were assured of the confidentiality of their personal information, and data were anonymized before analysis. Participation was voluntary, and participants had the right to withdraw at any point during the study. All patient data were handled in accordance with the principles of the Declaration of Helsinki.

### Data collection

Data were collected through structured interviews and self-administered questionnaires (**Supplementary File 1**) during the women’s first prenatal visit. The questionnaires were designed to gather comprehensive socio-demographic information and included questions on:

Maternal age at conception: The woman’s age at the point of conception.

Lifestyle factors: Smoking habits, alcohol consumption, and physical activity levels before pregnancy.

Health-related factors: Any chronic illnesses or past reproductive health issues.

Reproductive history: Any miscarriage or infertility treatments undertaken.

Marital status: The individual’s current relationship status, including whether she is single, married, or living with a partner.

Employment situation: The woman’s employment status, including whether she is employed full-time, part-time, or unemployed.

Highest educational attainment: The most advanced level of education completed by the individual, categorized as no formal education, primary, secondary, higher education, or postgraduate studies.

Income level: According to the economic standards of Chengdu, China, participants in this study were categorized into three income groups based on their monthly household income. Specifically, participants with a monthly income below 3000 RMB were classified as low-income, those with a monthly income between 3000 and 10000 RMB were classified as medium-income, and those with a monthly income above 10000 RMB were classified as high-income.

Furthermore, maternal health data was reviewed by analyzing medical records, which included details such as the gestational age at the initial prenatal visit and any complications occurring during the current or previous pregnancies. In this study, objective factors referred to measurable socio-demographic and health-related variables (e.g. age, BMI, education, income, employment, and reproductive health status), while subjective factors included participants’ self-reported perceptions, motivations, and psychological concerns regarding childbearing decisions. The classification of factors into subjective and objective categories was determined based on the inherent characteristics of the variables, not solely on the method of data collection.

### Statistical analysis

Descriptive statistical methods were used to summarize the demographic and socioeconomic characteristics of the participants. Continuous variables were presented as means and standard deviations, while categorical variables were presented as frequencies and percentages. Subsequently, binary logistic regression analysis was performed to identify the factors associated with postponed childbearing. The independent variables included in the regression model were selected based on factors identified in the literature as influencing postponed childbearing, as well as those variables that showed statistical significance in bivariate analysis. A *p* value of less than 0.05 was considered statistically significant. All statistical calculations were performed using SPSS software (version 26, IBM Corp.).

## Results

The selection process for the study began with a total of 1,207 pregnant women who were between 10 and 12 weeks of gestation. After excluding 26 women with a history of mental illness and 53 women who were unwilling to participate, the analytic sample consisted of 1,128 women. The sample was categorized by age: 507 women (44.9%) were aged 18–29 years, 453 women (40.2%) were aged 30–34 years, and 168 women (14.9%) were aged 35 years or older. Regarding subjective factors, 396 participants (35.1%) reported that psychological considerations influenced their decision to conceive, while 673 (59.7%) felt adequately prepared for childbirth (see Table 1).

**Table 1.** Description of the participant characteristics.

Variables	Total
Number	1128
Mean age (years)	29.73 ± 3.94
Pre-pregnancy BMI (kg/m^2^)	21.95 ± 2.87
Mode of conception	
Natural conception	1057 (93.7%)
Assisted reproductive technology	71 (6.3%)
Age stratification (years)	
18–29 years	507 (44.9%)
30–34 years	453 (40.2%)
≥35 years	168 (14.9%)
Marital status (married)	1025 (90.9%)
Educational levels	
No formal education, primary or secondary	159 (14.1%)
Tertiary education	758 (67.2%)
Postgraduate education	211 (18.7%)
Employment status	
Not employed	143 (12.7%)
Part-time or self-employed	202 (17.9%)
Full-time	783 (69.4%)
Income levels	
Low income	164 (14.5%)
Medium income	783 (69.4%)
High income	181 (16.0%)
Prepared for childbearing? (Yes)	673 (59.7%)

BMI body mass index.

### Objective factors associated with postponed childbearing

Univariate analysis revealed several factors associated with postponed childbearing (see Table 2). Women aged 30 and above were more likely to have attained a higher level of education and reported higher household income compared to their younger counterparts. Furthermore, full-time employment was significantly correlated with postponed childbearing. Additionally, the prevalence of reproductive health issues was found to be twice as high in women who experienced postponed childbearing compared to those in the normal childbearing group.

**Table 2.** Description of the participant characteristics by age types.

Variables	**Normal childbearing** *N* = 507	**Postponed childbearing** *N* = 621	*p* value
Age (years)	26.64 ± 3.78	32.25 ± 4.30	<0.001^a^
Mode of conception			0.088^b^
Natural conception	482 (95.1%)	575 (92.6%)	
Assisted reproductive technology	25 (4.9%)	46 (7.4%)	
Gravidity	1 (1)	2 (1)	0.686^c^
**Lifestyle factors**			
Pre-pregnancy BMI (kg/m^2^)	21.83 ± 2.97	22.04 ± 2.79	0.222^a^
Smoking	14 (2.8%)	23 (3.7%)	0.377^b^
Alcohol consumption history	27 (5.3%)	40 (6.4%)	0.430^b^
**Objective factors**			
Marital status (married)	462 (91.1%)	563 (90.7%)	0.788^b^
Educational levels			<0.001^b^
No formal education, primary or secondary	65 (12.8%)	94 (15.1%)	
Tertiary education	370 (73.0%)	388 (62.5%)	
Postgraduate education	72 (14.2%)	139 (22.4%)	
Employment status			<0.001^b^
Not employed	78 (15.4%)	65 (10.5%)	
Part-time or self-employed	131 (25.8%)	71 (11.4%)	
Full-time	298 (58.8%)	485 (78.1%)	
Income levels			<0.001^b^
Low-income	58 (11.4%)	106 (17.1%)	
Medium-income	387 (76.4%)	396 (63.8%)	
High-income	62 (12.2%)	119 (19.1%)	
Health-related factors			
Chronic conditions	31 (6.1%)	47 (7.6%)	0.338^b^
Reproductive health issues	34 (6.7%)	83 (13.4%)	<0.001^b^
**Subjective factors**			
Factors influencing reproductive intentions?			
Career advancement	308 (60.7%)	495 (79.7%)	<0.001^b^
Financial security	265 (52.2%)	423 (68.1%)	<0.001^b^
Suitable partner	159 (31.4%)	225 (36.2%)	0.086^b^
Psychological factors	168 (33.1%)	228 (36.7%)	0.497^b^
Prepared for Childbearing? (Yes)	264 (52.1%)	409 (65.9%)	<0.001^b^

BMI body mass index.

^a^ Average and standard deviation. Student’s *t* test.

^b^ Number (percentage). Chi-squared test.

^c^ Median (interquartile range). Kruskal–Wallis test.

**Figure 1.**
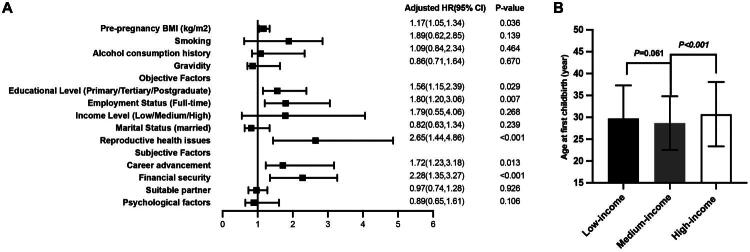
The impact of objective and subjective factors on postponed childbearing. A) Binary logistic regression analysis was conducted to further examine the impact of objective factors on postponed childbearing. After adjusting for covariates such as smoking, alcohol consumption history, gravidity, and subjective factors, the results indicated that pre-pregnancy BMI (β = 1.17, 95% CI: 1.05–1.34, *p* = 0.036), educational level (β = 1.56, 95% CI: 1.15–3.39, *p* = 0.029), employment status (β = 1.80, 95% CI: 1.20–3.06, *p* = 0.007), and reproductive health issues (β = 2.65, 95% CI: 1.44–4.86, *p* < 0.001) were revealed significant associations with postponed childbearing. Among subjective factors, career prioritization (OR = 1.72, 95% CI = 1.23–3.18, *p* = 0.013) and financial security (OR = 2.28, 95% CI = 1.35–3.27, *p* < 0.001) were the most significant predictors of postponed childbearing. B) Notably, subgroup analysis based on income level revealed that both low-income and high-income households were associated with postponed childbearing compared to medium-income households (low-income vs. medium-income, *p* = 0.041; medium-income vs. high-income, *p* < 0.001.

A binary logistic regression was performed to investigate the influence of objective factors on postponed childbearing (Figure 1A). After adjusting for potential confounders such as smoking, alcohol use history, number of pregnancies, and subjective variables, the analysis revealed that pre-pregnancy BMI (β = 1.17, 95% CI: 1.05–1.34, *p* = 0.036), educational attainment (β = 1.56, 95% CI: 1.15–3.39, *p* = 0.029), employment status (β = 1.80, 95% CI: 1.20–3.06, *p* = 0.007), and reproductive health concerns (β = 2.65, 95% CI: 1.44–4.86, *p* < 0.001) were significantly correlated with postponed childbearing. Specifically, each 1 kg/m^2^ increase in pre-pregnancy BMI corresponded to a 17% higher likelihood of postponed childbearing. Additionally, an increase in educational level was associated with a 60% higher risk of postponed childbearing. Reproductive health problems and full-time employment were found to elevate the risk by 1.65 and 0.80 times, respectively. Subgroup analysis based on income levels revealed that, compared to middle-income households, both low-income and high-income households are more likely to delay childbearing (middle-income vs. low-income, *p* = 0.041; middle-income vs. high-income, *p* < 0.001) (Figure 1B).

### Subjective factors influencing postponed childbearing

Subjective factors also played a significant role in delaying childbearing. A considerable portion of the participants, 71% (*n* = 803), identified career development as a primary reason, expressing a desire to secure professional achievements before starting a family. Financial stability was another essential consideration for 61% (*n* = 688) of women, who indicated that they preferred to reach a certain level of financial security prior to having children. Furthermore, the desire for a stable relationship was emphasized by 34% (*n* = 384) of the participants, who noted their inclination to wait until they felt more confident in their marital or partnership circumstances. Psychological concerns, including the fear of potential pregnancy complications and health risks related to advanced maternal age, were cited by 35% (*n* = 396) of women as significant influences on their reproductive choices (Table 1).

ChatGPT 说:

Among the factors identified, women in the postponed childbearing group were more frequently influenced by career progression and financial stability. Additionally, almost two-thirds of women in this category reported feeling adequately prepared for childbirth. A subsequent multivariate logistic regression analysis indicated that, in terms of subjective factors, prioritizing career (β = 1.72, 95% CI = 1.23–3.18, *p* = 0.013) and financial stability (β = 2.28, 95% CI = 1.35–3.27, *p* < 0.001) were the most significant determinants of postponed childbearing (Figure 1A). Women who placed career advancement first were 72% more likely to delay having children, whereas those focused on financial security were 128% more likely to postpone childbearing.

Notably, distinct psychological factors were identified between the normal and postponed childbearing groups. Women in the normal childbearing group primarily expressed concerns related to the challenges of child-rearing (*p* < 0.001), whereas those in the postponed childbearing group showed a stronger tendency to experience anxiety linked to advancing age (*p* < 0.001). Furthermore, individuals in the postponed childbearing group seemed to be more by societal and cultural pressures (see Table 3).

**Table 3.** Description of the psychological factors influencing participants’ reproductive intentions by age types.

Variables	Total	Normal childbearing group	Postponed childbearing group	*p* value^a^
Number	*N* = 1128	*N* = 507	*N* = 621	
Psychological factors				
Fear of parenting	107 (7.5%)	76 (15.0%)	31 (5.0%)	<0.001
Fear of maternal risks	81 (7.2%)	36 (7.1%)	45 (7.2%)	0.949
Age anxiety	136 (12.1%)	33 (6.5%)	103 (16.6%)	<0.001
Social and cultural expectations	54 (4.8%)	15 (3.0%)	39 (6.3%)	0.056
Others	18 (1.6%)	8(1.6%)	11(1.6%)	1.000

^a^ Number (percentage). Chi-squared test.

## Discussion

This study aimed to analyze the factors influencing postponed childbearing in women, focusing on both subjective and objective factors. The findings revealed that women in the postponed childbearing group were significantly influenced by a combination of health, socio-economic, and psychological factors, compared to women in the normal childbearing group. These results suggest that the decision to delay childbirth is multifactorial, involving both external pressures and internal perceptions.

### Objective factors and their role in postponed childbearing

Consistent with previous research, our study found that objective factors such as pre-pregnancy BMI, educational level, employment status, and reproductive health issues were key contributors to postponed childbearing [21]. Specifically, higher pre-pregnancy BMI was associated with an increased risk of postponed childbirth. This finding is in line with studies indicating that obesity can impair fertility, leading women to delay childbirth while seeking solutions to manage their health or undergo fertility treatments [22].

Educational level also played a significant role in the decision to delay childbirth. Women with higher educational attainment were more likely to delay childbearing. This is consistent with the notion that higher education is often correlated with career development and economic stability, both of which may lead to postponement of pregnancy [21,23]. Similarly, full-time employment was associated with postponed childbearing, with women in full-time employment being more likely to delay childbirth. This could reflect the prioritization of career advancement, which often results in postponed family formation.

Another significant objective factor identified was the presence of reproductive health issues, which doubled the likelihood of postponed childbearing. This finding underscores the impact of reproductive health challenges on women’s fertility decisions [24]. Women with a history of reproductive health issues may delay childbirth in hopes of improving their health or utilizing assisted reproductive technologies when they feel better prepared or more financially stable.

### Subjective factors and their influence on childbearing decisions

In terms of subjective factors, the study highlighted the strong influence of psychological factors on the decision to delay childbearing. Women in the postponed childbearing group reported that career advancement and financial security were the most significant predictors of postponed childbirth. This reflects the growing trend of women balancing their professional and personal lives before making the decision to have children.

It is also noteworthy that nearly two-thirds of the women in the postponed childbearing group felt that they were prepared for childbirth. However, the decision to delay seemed to be driven more by external factors, such as career goals and financial stability, than by a lack of preparedness for motherhood. This aligns with previous findings that women often delay childbearing due to perceived external pressures, such as the need to establish financial stability and achieve career goals [25].

Furthermore, the analysis revealed that women in the postponed childbearing group experienced anxiety related to age, while women in the Normal Childbearing group were primarily concerned with the fear of raising children. The anxiety about age-related fertility issues in postponed childbearing women has been well documented in the literature, and it is consistent with the growing awareness of the reproductive age [26]. The fear of raising children, on the other hand, was more prevalent in younger women, highlighting the psychological burden of early childbearing [27].

Moreover, the influence of social and cultural expectations was more pronounced among women in the postponed childbearing group. Cultural norms, family pressures, and societal expectations around motherhood can significantly impact women’s reproductive decisions. The decision to delay childbearing may not only be influenced by personal preferences but also by societal values and the increasing focus on women’s roles in the workforce and as individuals.

### Subgroup analysis based on income level

One of the most striking findings of this study was the role of income in postponed childbearing. Analysis of subgroups by income levels indicated that postponed childbearing was linked to both low-income and high-income households when compared to those with medium-income. This result suggests that economic factors, including financial stability and access to resources, play a critical role in women’s reproductive decisions [28]. Women from low-income households may delay childbearing due to concerns about the affordability of raising children, while women from high-income households may delay in order to maintain their career trajectory or achieve greater economic security before starting a family.

### Integrating subjective and objective factors: a multifactorial approach

Our study clearly demonstrates that postponed childbearing is not attributable to any single factor. Rather, it is the result of a complex interaction between both subjective and objective influences. While health-related and socio-economic factors set the stage for postponed childbearing, it is subjective elements, such as psychological concerns, career aspirations, and financial security, that often drive the timing of childbirth [19]. Women in our study, particularly those in the postponed childbearing group, were not only influenced by their biological and economic circumstances but also by the psychological and cultural frameworks within which they operate.

This multifactorial approach is important for understanding postponed childbearing and suggests that interventions to support women in making reproductive decisions must consider both the objective barriers and the subjective perceptions they face. Public health initiatives could therefore benefit from addressing both the tangible and intangible aspects of childbearing decisions. For example, policies aimed at supporting women in balancing career and family life, such as parental leave and workplace flexibility, could help alleviate the socio-economic pressures that contribute to postponed childbearing. Additionally, psychological support, including fertility counseling, could help address concerns about age and reproductive health, empowering women to make informed decisions about the timing of motherhood [27].

### Implications for public health and future research

The findings of this study have significant implications for public health policy and interventions aimed at supporting women’s reproductive health. While many women may feel that delaying childbearing is a rational and necessary decision based on career and financial factors, it is crucial that they have access to comprehensive reproductive health care, including education about fertility, the risks of delaying childbearing, and the availability of assisted reproductive technology options.

Despite the strengths of this study, there are several limitations to acknowledge. The cross-sectional nature of the design prevents the establishment of causal relationships, and the use of self-reported data may lead to potential biases, including recall bias or social desirability bias. The study’s regional focus may limit its global applicability, and unmeasured variables such as partner influence and healthcare access could further inform the findings. Furthermore, by including only women who were able to conceive, the study may have overlooked factors affecting women who remain childless. These limitations underscore the need for longitudinal and more inclusive studies to validate and expand upon the current findings. Future research should further explore the dynamic relationship between subjective and objective factors and how these factors evolve over time. Longitudinal studies that track women’s reproductive health, career trajectories, and psychological wellbeing could provide deeper insights into the processes leading to postponed childbearing. In addition, broadening the sample to encompass women from a variety of cultural, ethnic, and socio-economic groups would aid in determining whether these factors have consistent effects across different populations.

## Conclusion

To summarize, this research underscores the intricate relationship between both subjective and objective factors that shape the decision to delay childbearing in women. Notable objective factors, including pre-pregnancy BMI, level of education, employment status, and reproductive health concerns, were found to play a crucial role in the postponement of childbirth. Subjectively, career prioritization, financial security concerns, and psychological factors such as age-related anxiety played a prominent role in women’s reproductive decisions. Income level was shown to play a role in the timing of childbirth, with both lower- and higher-income women exhibiting a greater tendency to postpone having children. These results highlight the complex factors contributing to postponed childbearing, stressing the importance of policies that consider the health and socio-economic difficulties women encounter, while providing support in areas such as career development, family life, and reproductive health.

## Supplementary Material

Supplemental Material

## Data Availability

Data are available upon reasonable request to the corresponding author.
