# Supplementary material for: Postponed childbearing: a cross-sectional study of differences between subjective and objective factors
Source: Ann Med. 2025 Aug 27;57(1):2546674. doi: 10.1080/07853890.2025.2546674 (PMC12395614; doi:10.1080/07853890.2025.2546674)
Supplement: Supplemental Material [file IANN_A_2546674_SM0138.docx]

# **Questionnaires for fertility-related mental health status and social stress**

# Participant overview

Participant ID:

Center:

Midwife:

Registration date:

Recruitment date:

## Recruitment

### 1. Registeration

* Participant ID： * Participant’s name： *MW Contact Date： *Study ID：

### 2. Agreement

*I agree to take part in this study. Yes NO

1. I have been reading the informed consent, the researchers have explained the purpose, contents, risks and benefits of this research to me clearly. My questions so far have been answered. I understand the information printed on this form, and I volunteered for this study.

Yes NO

1. I agree to donate my biological samples (including blood, urine, hair, nails, buccal smear) and my baby's biological samples and my husband's oral samples for this research. I know our biological samples such as blood, urine and hair could be sent to study abroad, and I know the donation is voluntary. I could withdraw from the study whenever I decide, which won’t affect the normal antenatal care in the Chengdu Women's and Children's Central Hospital.

Yes NO

3. I agree the researchers to check on my medical records and personal information related to medicine. I know that my personal information will be kept secret. Yes NO

4. I agreed to the use of biological samples and personal information I donated in current and future scientific research (including commercial research and scientific research not related to this project). I know clearly that if this research could lead to new treatments and inventions of medical testing, I would not be able to gain commercial benefits from it.

Yes NO

5. I have the opportunity to invite my family or friends to help me to ask questions about this study, I know the person I should contact with if there is a problem. Yes NO

### 3. Enrolment

Group： ①Age ＜ 30 ②Age (30-35) ③Age ≥ 35

Inclusion Criteria：* Singleton pregnancy

* Gestational age of 10 to 12 weeks

***** Able to provide written, informed consent

Exclusion Criteria: ***** Severe mental illnesses, such as schizophrenia, bipolar disorder, or major

depressive disorder requiring hospitalization

***** Unwilling to participate

### 4. Pregnant woman basic information

*name：

*date of birth：

*native place：

*Telephone number：

*address：

* Patient ID：

*Prenatal examination file number：

*education：1- primary school 2- high school 3- University 4- university or above 5- illiterate 6- other:

*occupation：

*income (per month)：

*employment status： (Not employed/Part-time or self-employed/Full-time)

*blood type：

*height： cm

*weight before pregancy： kg

*smoke（1-no 2- smoking before 3-still smoking, /d 4-History of passive smoking, years） *alcohol（1-no 2- drinking before 3-still drinking, g/week）

*husband’s name：

*age：

*height： cm

*blood type：

*education：1- primary school 2- high school 3- University 4- university or above 5- illiterate 6- other:

*occupation：

*income (per month)：

*telephone number：

### 5. Obstetric History

*pregnancy（times）：_______（Including pregnancy, termination of pregnancy, abortion）

*delivery（times）：_______

* fetal malformations：①yes（add time）②no

* premature delivery：①yes（add time） ②no

*Stillbirth：①yes（add time） ②no

*tractus genitalis operation history（Cesarean section、obstetric forceps）：①yes（add time） ②no *spontaneous abortion：①yes（add time）②no

*artificial abortion：①yes（add time） ②no

*odinopoeia：①yes（add time） ②no

*acephalocystis racemosa：①yes（add time） ②no

* Gestational diabetes mellitus：①yes（add time） ②no

* intrahepatic cholestasis of pregnancy：①yes（add time） ②no

* Pre-eclampsia：①yes（add time） ②no

### 6. Current Pregnancy

* Last menstrual period：

* Gestational weeks：

*pregnancy mode：① Natural pregnancy ②IVF-ET ③ovulation stimulants

### 7. History of Present Illness

*colporrhagia：①yes ②no

*Viral infection：①yes ②no

*Exposure to radiation：①yes ②no

*hypermesis：①yes ②no

*fever：①yes ②no

* Long term exposure to poison：①yes ②no

* take medicine：①yes (add drug name and time) ②no

* anemia：①yes(add type) ②no

* take acyeterion before pregnancy 6 months：①yes（add drug name） ②no

other：

### 8. Past medical history

*heart disease：①yes ②no

*high blood pressure：①yes ②no

*nephritis：①yes ②no

*hepatitis：①yes ②no

*Tuberculosis：①yes ②no

* diabetes mellitus：①yes ②no

*blood disease：①yes ②no

* Mental illness：①yes (add type) ②no

*epilepsy：①yes ②no

*thyroid dysfunction：①yes ②no

*allergic history**：**①yes ②no

* operation history：①yes（add operation name and time） ②no

other：

### 9. Family History

*Twin history：①yes（add anyone）②no

* Gestational diabetes mellitus：①yes（add anyone）②no

* intrahepatic cholestasis of pregnancy：①yes（add anyone）②no

* Pre eclampsia：①yes（add anyone）②no

*neuropathy：①yes（add anyone）②no

*dementia：①yes（add anyone）②no

*malformation：①yes（add anyone）②no

*genetic disease：①yes（add anyone）②no

*high blood pressure：①yes（mother or father） ②no

* diabetes mellitus：①yes（mother or father） ②no

*Cardiovascular diseases：①yes（mother or father） ②no

* Immune diseases：①yes（mother or father） ②no

* Obstetric History：①yes（mother） ②no

*tumor history：①yes（mother or father） ②no

other：

### Psychosocial Factors Items

| Factor | Effect | Sample Questionnaire Items |
| --- | --- | --- |
| Career Advancement | Yes /No | - “I prefer to establish my career before having children.” - “Having a child may interfere with my professional development.” |
| Fear of Parenting | Yes /No | - “I am concerned I may not be able to raise a child well.” - “I worry about the emotional and physical burden of parenting.” |
| Fear of Financial Insecurity | Yes /No | - “I want to be financially stable before becoming a parent.”  - “I fear that having a child would lead to financial difficulties.” |
| Age-related Anxiety | Yes /No | - “I am anxious about the risks of pregnancy as I get older.” - “I worry that waiting longer may reduce my chances of a healthy pregnancy.” |
| Partner Factors | Yes /No | - “My partner prefers to delay having children due to career or financial concerns.” - “My partner and I have not reached a consensus on when to start a family.” |
| Social and Cultural Expectations | Yes /No | - “My parents expect me to have children before a certain age.”  - “I feel social pressure to follow traditional timelines for marriage and childbearing.” |

## Edinburgh Postnatal Depression Scale (EPDS)

The EPDS is a 10-item self-report scale designed to screen women for symptoms of emotional distress during the postnatal period. Respondents are asked to select one of four responses that best describes how they have felt during the past 7 days.

1. I have been able to laugh and see the funny side of things.

2. I have looked forward with enjoyment to things.

3. I have blamed myself unnecessarily when things went wrong.

4. I have been anxious or worried for no good reason.

5. I have felt scared or panicky for no very good reason.

6. Things have been getting on top of me.

7. I have been so unhappy that I have had difficulty sleeping.

8. I have felt sad or miserable.

9. I have been so unhappy that I have been crying.

10. The thought of harming myself has occurred to me.

## 12. Perceived Stress Scale (PSS-10)

The PSS-10 is a 10-item instrument designed to measure the perception of stress. Each item is rated on a 5-point Likert scale from 0 (never) to 4 (very often), based on experiences during the last month.

1. 1. In the last month, how often have you been upset because something that happened unexpectedly?
2. 2. In the last month, how often have you felt that you were unable to control the important things in your life?
3. 3. In the last month, how often have you felt nervous and 'stressed'?
4. 4. In the last month, how often have you felt confident about your ability to handle your personal problems?
5. 5. In the last month, how often have you felt that things were going your way?
6. 6. In the last month, how often have you found that you could not cope with all the things that you had to do?
7. 7. In the last month, how often have you been able to control irritations in your life?
8. 8. In the last month, how often have you felt that you were on top of things?
9. 9. In the last month, how often have you been angered because of things that were outside of your control?
10. 10. In the last month, how often have you felt difficulties were piling up so high that you could not overcome them?

## State-Trait Anxiety Inventory (STAI)

The STAI consists of two subscales of 20 items each: State Anxiety (Form Y-1) and Trait Anxiety (Form Y-2). Each item is rated on a 4-point scale. State anxiety items assess how the respondent feels 'right now, at this moment', while trait anxiety items assess how the respondent generally feels.

### State Anxiety (Form Y-1)

1. 1. I feel calm.
2. 2. I feel secure.
3. 3. I am tense.
4. 4. I feel strained.
5. 5. I feel at ease.
6. 6. I feel upset.
7. 7. I am presently worrying over possible misfortunes.
8. 8. I feel satisfied.
9. 9. I feel frightened.
10. 10. I feel comfortable.
11. 11. I feel self-confident.
12. 12. I feel nervous.
13. 13. I am jittery.
14. 14. I feel indecisive.
15. 15. I am relaxed.
16. 16. I feel content.
17. 17. I am worried.
18. 18. I feel confused.
19. 19. I feel steady.
20. 20. I feel pleasant.

### Trait Anxiety (Form Y-2)

1. 1. I feel pleasant.
2. 2. I tire quickly.
3. 3. I feel like a failure.
4. 4. I feel rested.
5. 5. I am 'calm, cool, and collected'.
6. 6. I feel that difficulties are piling up so that I cannot overcome them.
7. 7. I worry too much over something that really doesn’t matter.
8. 8. I am happy.
9. 9. I have disturbing thoughts.
10. 10. I lack self-confidence.
11. 11. I feel secure.
12. 12. I make decisions easily.
13. 13. I feel inadequate.
14. 14. I am content.
15. 15. Some unimportant thought runs through my mind and bothers me.
16. 16. I take disappointments so keenly that I can’t put them out of my mind.
17. 17. I am a steady person.
18. 18. I get in a state of tension or turmoil as I think over my recent concerns and interests.
19. 19. I feel satisfied with myself.
20. 20. I have trouble sleeping because of worries.
